# Sustained HIV Suppression With Co-formulated Tenofovir Disoproxil Fumarate/Lamivudine/Dolutegravir in a Person With Transmitted Dolutegravir Resistance and Pretreatment Resistance to Lamivudine: a Case Report From HPTN 083

**DOI:** 10.1093/ofid/ofaf645

**Published:** 2025-10-15

**Authors:** Hannah P Moore, Jessica M Fogel, Mark A Marzinke, Elias K Halvas, Urvi M Parikh, John W Mellors, Kerri J Penrose, Michael Seisa, Christos Petropoulos, Amelia L Price, Amber Moser, Zhe Wang, Marybeth McCauley, Javier Valencia-Huamaní, Alex R Rinehart, James F Rooney, Lydia Soto-Torres, Beatriz Grinsztejn, Raphael J Landovitz, Susan H Eshleman

**Affiliations:** Department of Pathology, Johns Hopkins University School of Medicine, Baltimore, Maryland, USA; Department of Pathology, Johns Hopkins University School of Medicine, Baltimore, Maryland, USA; Department of Pathology, Johns Hopkins University School of Medicine, Baltimore, Maryland, USA; Department of Medicine, Johns Hopkins University School of Medicine, Baltimore, Maryland, USA; Department of Medicine, University of Pittsburgh, Pittsburgh, Pennsylvania, USA; Department of Medicine, University of Pittsburgh, Pittsburgh, Pennsylvania, USA; Department of Medicine, University of Pittsburgh, Pittsburgh, Pennsylvania, USA; Department of Medicine, University of Pittsburgh, Pittsburgh, Pennsylvania, USA; Monogram Biosciences, South San Francisco, California, USA; Monogram Biosciences, South San Francisco, California, USA; Department of Medicine, Johns Hopkins University School of Medicine, Baltimore, Maryland, USA; Department of Pathology, Johns Hopkins University School of Medicine, Baltimore, Maryland, USA; Statistical Center for HIV/AIDS Research and Prevention, Vaccine and Infectious Disease Division, Fred Hutchinson Cancer Center, Seattle, Washington, USA; FHI 360, Durham, North Carolina, USA; Asociacion Civil Impacta Salud y Educacion, Lima, Peru; ViiV Healthcare, Research Triangle Park, Durham, North Carolina, USA; Gilead Sciences, Foster City, California, USA; Prevention Science Program, Division of AIDS, National Institute of Allergy and Infectious Diseases, National Institutes of Health, Rockville, Maryland, USA; Instituto de Pesquisa Clinica Evandro Chagas-Fiocruz, Rio de Janeiro, Brazil; Center for Clinical AIDS Research and Education, University of California, Los Angeles, Los Angeles, California, USA; Department of Pathology, Johns Hopkins University School of Medicine, Baltimore, Maryland, USA

**Keywords:** drug resistance, HIV, INSTI, TLD, transmitted

## Abstract

Integrase strand transfer inhibitors (INSTIs) are recommended in most first-line HIV treatment regimens. We describe a participant in a clinical trial with transmitted INSTI resistance. The participant had no history of INSTI use and had no evidence of INSTI exposure prior to HIV acquisition. Treatment with tenofovir disoproxil fumarate, lamivudine (3TC), and dolutegravir (DTG) was started 3 weeks after HIV diagnosis. Viral suppression was achieved within a year and was sustained for >3 years on treatment. Retrospective HIV genotyping of a pretreatment sample detected major resistance mutations in 3 drug classes, with predicted high-level resistance to DTG and 3TC. HIV phenotyping confirmed that the transmitted virus had DTG and 3TC resistance but retained susceptibility to DTG at higher drug concentrations. Pharmacologic testing indicated that the DTG concentrations observed in this case were sufficient to overcome the effects of 2 major baseline INSTI resistance mutations (G140S and Q148H).

Integrase strand transfer inhibitors (INSTIs) are included in most first-line regimens recommended globally for HIV antiretroviral treatment (ART) [1]. Despite the widespread use of INSTI-based ART, the prevalence of INSTI resistance remains low [2]. Studies of persons with HIV who are newly diagnosed or ART-naïve suggest that transmission of INSTI-resistant HIV is uncommon [3–8]. To date, there is limited information characterizing individual cases of transmitted INSTI resistance and the response to INSTI-based ART in those cases.

The HIV Prevention Trials Network (HPTN) 083 trial compared the efficacy of a long-acting injectable INSTI, cabotegravir (CAB-LA), to daily oral tenofovir disoproxil fumarate (TDF)/emtricitabine (FTC) for HIV pre-exposure prophylaxis (PrEP) [9]. The blinded portion of the study was stopped early by an independent safety monitoring board for a finding of superiority of CAB-LA [9]. In HPTN 083, INSTI resistance-associated mutations (RAMs) were observed in most of the participants in the CAB-LA arm who acquired HIV-1 near the time of CAB-LA injections [10, 11]. In this report, we describe a participant in HPTN 083 who did not receive cabotegravir (CAB) and had transmitted multi-class resistance detected by retrospective testing that included resistance to dolutegravir (DTG) and lamivudine (3TC). The report includes an evaluation of the participant's response to ART with DTG, 3TC, and TDF (TLD ART) over more than 3 years of follow-up.

## CASE PRESENTATION

The HPTN 083 trial (ClinicalTrials.gov #NCT02720094) enrolled cisgender men and transgender women who have sex with men and who did not have HIV. Participants were randomized 1:1 to receive CAB-LA or daily oral TDF/FTC. In the blinded phase of the trial, participants in the TDF/FTC arm received oral TDF/FTC and placebo CAB tablets and injections. Previous reports describe the study design and outcomes of the HPTN 083 trial [9, 12]. Laboratory methods used to evaluate the case in this report are presented in Supplementary File 1.

**Figure 1.**
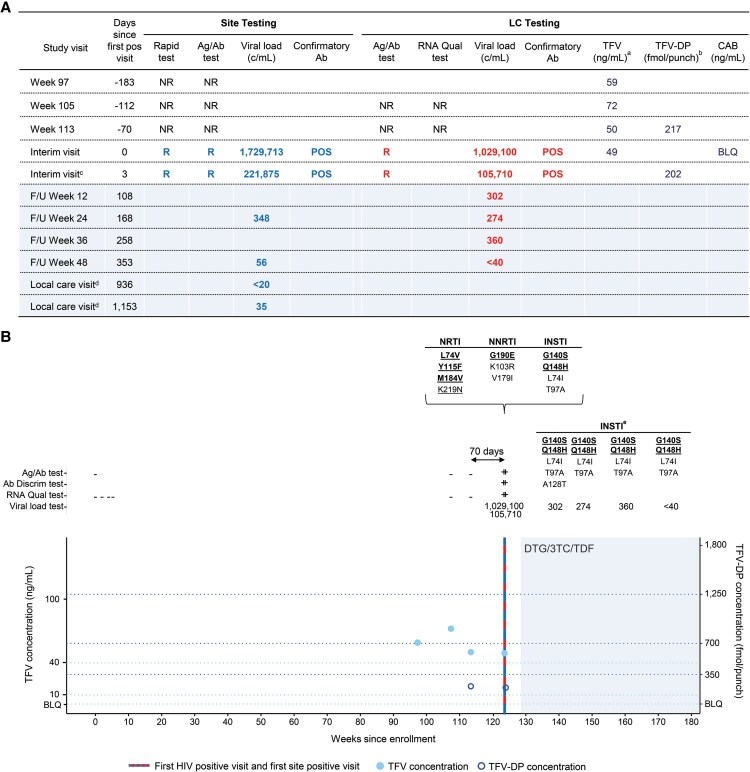
Case characteristics. The figure shows clinical events and laboratory results for the participant described in this report. *A*, Results in the table are shown for real-time testing performed at the study site (Site Testing) and retrospective testing performed at the HPTN LC (LC Testing), starting with the Week 97 visit. Results from site-based HIV rapid testing and Ag/Ab testing at all prior visits were non-reactive. Reactive/positive results are shown in bold (blue font: Site Testing; red font: LC Testing). Blue shading indicates visits when the participant was on antiretroviral treatment (ART). *B*, Annotations above the graph show results obtained from retrospective testing performed at the HPTN LC. A plus sign (+) indicates a reactive or positive test result. A minus sign (−) indicates a non-reactive or negative test result. Viral load values show the number of HIV RNA copies/mL. Resistance-associated mutations (RAMs) are shown above the graph; major RAMs are shown in bold. Mutations recommended for surveillance of transmitted drug resistance are underlined. Blue shading indicates visits when the participant was on ART. ^a^TFV concentrations of 10 and 40 ng/mL correspond to 4 and 7 doses/week, respectively [13]. ^b^TFV-DP concentrations of 350, 700, and 1250 fmol/punch correspond to 2, 4, and 7 doses/week, respectively [14]. ^c^The participant started ART 21 d later with tenofovir disoproxil fumarate, lamivudine, and dolutegravir (TLD). Blue shading indicates visits when the participant was on ART. ^d^The viral load data from local care visits were obtained from review of laboratory reports and the standard local care medical record. The participant remained on TLD ART after leaving the HPTN 083 study and reported good adherence to the ART regimen (personal communication, J. Valencia-Huamaní). ^e^Resistance was evaluated using a single-genome sequencing assay for samples with viral loads <500 copies/mL (integrase region only). Abbreviations: 3TC, lamivudine; Ab, antibody; Ag/Ab test, instrumented antigen/antibody test; BLQ, below limit of quantification; CAB, cabotegravir; c/mL, copies/milliliter; Discrim, discriminatory; DTG, dolutegravir; fmol, femtomole; F/U, follow-up visit; INSTI, integrase strand transfer inhibitor; LC, (HPTN) Laboratory Center; NNRTI, non-nucleoside reverse transcriptase inhibitor; NR, non-reactive; NRTI, nucleoside/nucleotide reverse transcriptase inhibitor; pos, positive; Qual, qualitative; R, reactive; TDF, tenofovir disoproxil fumarate; TFV, tenofovir; TFV-DP, tenofovir diphosphate.

The participant described in this report was randomized to receive daily oral TDF/FTC and received blinded study product for 14 months. After the HPTN 083 trial was unblinded in May 2020, the participant received open-label TDF/FTC for an additional 19 months. The first visit with documented HIV infection (first HIV-positive visit) was 123 weeks after study enrollment and 64 weeks after the last placebo injection (Figure 1). At that visit, HIV test results obtained at the study site included a reactive HIV rapid test, a reactive laboratory-based antigen/antibody test, a positive HIV antibody discriminatory test, and a positive HIV RNA test (1 729 713 copies/mL; Figure 1). These results were confirmed by retrospective testing at the HPTN Laboratory Center; HIV RNA was not detected at the 2 prior visits (Figure 1). Adherence to daily oral TDF/FTC was evaluated by quantifying tenofovir (TFV) in plasma and tenofovir diphosphate (TFV-DP) in dried blood spots (DBS) (Supplementary File 1). At steady state, the look-back windows for plasma TFV and DBS TFV-DP are approximately 7–10 days and 8 weeks, respectively [15, 16]. The plasma TFV concentration at the first HIV-positive visit (49 ng/mL) was consistent with daily TDF/FTC adherence or recent drug exposure. However, the low concentration of TFV-DP 3 days later (202 fmol/punch) suggested that the participant had poor or intermittent adherence in the prior 8 weeks [9, 17] (Figure 1). Similar discrepancies between plasma TFV and DBS TFV-DP concentrations were observed in the preceding months. To confirm that CAB was not administered to the participant in error, plasma from the first HIV-positive visit was tested for CAB; CAB was not quantifiable in that sample (Figure 1). HIV genotyping was not performed at the study site prior to ART initiation in accordance with the standard of care at the study site. ART was started 3 weeks after the first HIV-positive visit with a once-daily single-tablet regimen of TDF (300 mg), 3TC (300 mg), and DTG (50 mg; TLD regimen). The viral load dropped from over one million copies/mL to 302 copies/mL in 3 months and was <40 copies/mL after 11 months of ART (Figure 1). The participant remained on TLD with sustained viral suppression (viral load <40 copies/mL) for more than 2 years after study discontinuation (more than 3 years on ART).

Retrospective HIV genotyping was performed using a commercial genotyping assay based on next-generation sequencing (GenoSure PRIme) (Supplementary File 1) [18]. This analysis revealed that the participant had multi-class resistance at the first HIV-positive visit (Figure 1), with RAMs for 3 drug classes (INSTIs, non-nucleoside reverse transcriptase inhibitors [NNRTIs], and nucleoside/nucleotide reverse transcriptase inhibitors [NRTIs]). Phylogenetic analysis revealed that the participant had subtype B HIV. Two major INSTI RAMs (G140S, Q148H), one major NNRTI RAM (G190E), and 3 major NRTI RAMs (L74V, Y115F, M184V) were detected at that visit, along with accessory RAMs for all 3 drug classes. All of the major RAMs and one of the accessory RAMs (K219N) detected are among the mutations recommended for surveillance of transmitted drug resistance [19, 20]. None of the RAMs or other mutations detected at the first HIV-positive visit were reported as mixtures.

A low viral load single-genome sequencing assay (integrase region only) [21, 22] was used to assess INSTI resistance in samples collected after ART initiation; 70 single-genome sequences were generated from samples from 4 study visits spanning 245 days. The INSTI RAMs detected at the first HIV-positive visit were present as linked RAMs in all 70 single-genome sequences (Supplementary File 2). The RAMs detected in the pre-ART and on-ART samples predicted high-level resistance to 5 INSTIs (bictegravir [BIC], CAB, DTG, elvitegravir, raltegravir), 4 NNRTIs (doravirine, efavirenz, nevirapine, rilpivirine), and 4 NRTIs (3TC, abacavir [ABC], didanosine, emtricitabine) [23].

**Figure 2.**
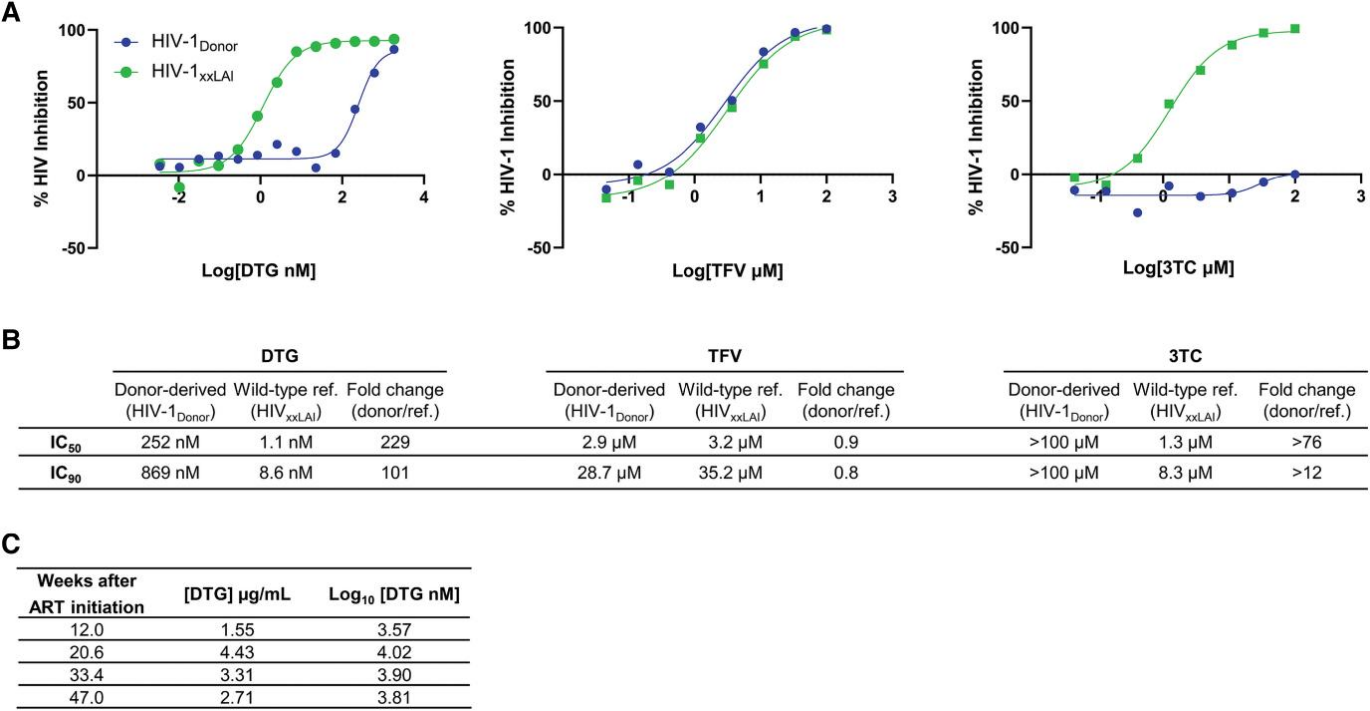
Phenotypic and pharmacologic testing. *A*, This panel shows the results from phenotypic drug testing for dolutegravir (DTG), tenofovir (TFV), and lamivudine (3TC) using a sample from the participant's first HIV-positive visit. Methods used for testing are described in Supplementary File 1. Results obtained for the donor-derived virus are shown in blue. Results obtained for the wild-type reference virus (HIVxxLAI) are shown in green. The X-axis shows the log of drug concentration and the Y-axis shows the percentage of inhibition of HIV replication. HIV phenotyping results for additional drugs are shown in Supplementary File 3; note that additional drug concentrations were tested for DTG compared to other drugs. *B*, This table shows the *in vitro* 50% and 90% inhibitory concentrations (IC_50_ and IC_90_ values) and the fold-change values for drug susceptibility for the donor-derived virus and the wild-type reference virus. *C*, This panel shows the DTG concentration in 4 samples collected from the participant after initiation of antiretroviral therapy with tenofovir disoproxil fumarate (TDF), 3TC and DTG (TLD). Note that the timing of collection of study samples relative to the timing of dosing of TLD is not known. Abbreviations: 3TC, lamivudine; ART, antiretroviral therapy; DTG, dolutegravir; IC_50_, *in vitro* 50% inhibitory concentration; IC_90_, *in vitro* 90% inhibitory concentration; ref., reference; TFV, tenofovir.

Phenotypic resistance testing confirmed that the virus had resistance to 2 of the 3 drugs in the ART regimen (DTG and 3TC), without resistance to TFV at the first HIV-positive visit (Figure 2); resistance was also observed for the INSTIs BIC and CAB (Supplementary File 3). The fold change in DTG concentration required for 90% inhibition of viral replication (IC_90_) for the donor virus compared to the reference virus was 101 (Figure 2). The DTG predicted protein-adjusted IC_90_ for the donor-derived virus and wild-type reference virus were 15 294 nM (4.18 log_10_) and 152 nM (2.18 log_10_), respectively. Despite this high level of DTG resistance, viral suppression was achieved at higher DTG concentrations *in vitro*. DTG concentrations were tested in on-ART samples from the study participant. In 3 of the 4 samples, DTG concentrations were between the reported geometric mean C_min_ and C_max_ for TLD (C_min_ = 1.11 µg/mL = 3.42 log_10_ [DTG nM]; C_max_ = 3.67 µg/mL = 3.94 log_10_ [DTG nM]) [24]; the DTG concentration in the fourth sample was above the C_max_ (Figure 2).

## DISCUSSION AND CONCLUSIONS

In this case report, viral suppression was achieved with TLD ART within 11 months of ART initiation and was sustained for more than 3 years of follow-up despite high-level pretreatment genotypic resistance to both DTG and 3TC. The participant had HIV with major RAMs that predicted INSTI, NRTI, and NNRTI resistance. None of the RAMs were detected as mixtures in the GenoSure PRIme assay, and both major INSTI RAMs were present in all single-genome sequences. These findings indicate that all major RAMs detected were present in the same viral genomes. The participant did not receive CAB in the trial and CAB was not quantifiable in plasma at the first HIV-positive visit. The participant also did not have any known exposure to NNRTIs or other INSTIs. The time between the last HIV-negative visit and the first HIV-positive visit was relatively short (70 days); the viral load was also very high at the first HIV-positive visit (>1 million copies/mL). These findings suggest that HIV acquisition likely occurred shortly before the first HIV-positive visit. For these reasons, it is highly likely that the resistance to NNRTIs and INSTIs was transmitted. The participant was receiving TDF/FTC in the trial and pharmacologic testing indicated that the participant was taking TDF/FTC, even though drug concentrations were not consistent with daily adherence. It was not possible to determine if the NRTI RAM M184V was transmitted or was rapidly selected after HIV acquisition by the oral PrEP drugs. The other 2 NRTI RAMs detected, L74V and Y115F, are commonly selected by ABC but are only rarely selected by TFV-containing regimens [23]. K219N is an accessory thymidine analog mutation (TAM) not associated with TDF/FTC use and usually occurs with multiple other TAMs [23]. These findings suggest that most of the NRTI RAMs detected (L74V, Y115F, K219N) were also transmitted.

The participant started TLD ART 3 weeks after HIV diagnosis. Baseline HIV genotyping was not performed at the study site, consistent with local guidelines. Despite having high-level resistance to 2 of the 3 drugs in the treatment regimen (DTG and 3TC) prior to ART initiation, the participant had a significant decline in HIV viral load after treatment initiation, achieved viral suppression with a viral load below 40 copies/mL within 11 months, and remained virally suppressed on TLD for more than 3 years. The favorable response to TLD ART in this case was likely explained by the high DTG concentrations in plasma that were achieved with the TLD regimen. These concentrations were close to the predicted concentration of DTG required for 90% inhibition of the virus *in vivo* [25]. The presence of the M184V mutation may also have increased susceptibility to TDF [26]; this mutation has been shown to reduce the fitness of some HIV viruses [27].

In 2018, the World Health Organization recommended DTG-based ART as the preferred first- and second-line regimen for treatment of children and adults with HIV [1]. Resistance testing is not feasible or affordable in some low- and middle-income countries and individuals often initiate DTG-based ART without resistance testing. Current guidelines in the United States (US) recommend genotypic resistance testing of HIV reverse transcriptase and protease prior to ART initiation [28, 29]. Baseline resistance testing of HIV integrase is only recommended for individuals with suspected transmitted INSTI resistance, prior use of CAB-LA for PrEP, or prior use of INSTIs for post-exposure prophylaxis [28, 29]. Data supporting baseline integrase genotyping in cases of suspected transmitted INSTI resistance are limited. While recent studies have evaluated transmitted INSTI resistance [3–8], ART outcomes were not assessed for individuals with INSTI resistance in those studies.

Response to DTG-based ART has also been evaluated in persons who acquired INSTI resistance from prior HIV treatment. The VIKING-3 study evaluated persons who were failing raltegravir- or elvitegravir-based ART with INSTI resistance who transitioned to DTG-based ART [30]. More than two thirds of the participants achieved viral suppression within 24 weeks of ART initiation. In that study, baseline INSTI resistance was predictive of the response to DTG-based ART; individuals who had a Q148 mutation with 2 or more INSTI RAMs (G140A/C/S, E138A/K/T, or L74I) at baseline were less likely to respond to ART within 24 weeks. The participant in this report had Q148H with 2 of the other INSTI RAMs noted (G140S and L74I).

To our knowledge, this is the first reported case of transmitted INSTI resistance that includes information about virologic suppression on DTG-based ART. The findings from retrospective phenotypic resistance testing and pharmacologic drug testing in this case demonstrate that transmitted HIV with major INSTI RAMs may retain susceptibility to DTG at drug concentrations attained with routine TLD treatment. In this case, if HIV genotyping had been performed prior to ART initiation, the results would likely have discouraged use of INSTI-based ART. Phenotypic DTG susceptibility was only observed in this case at drug concentrations that were near the maximum concentration obtained *in vivo* with 50 mg daily dosing (C_max_ = 3.67 µg/mL = 3.94 log_10_ [DTG nM]); however, as the timing of TLD dosing is unknown, full pharmacologic inferences cannot be made [24]. Lapses in adherence to the treatment regimen or use of concomitant medications known to interact with DTG could potentially compromise DTG effectiveness.

The 2024 WHO HIV drug resistance report found that the prevalence of DTG resistance in some regions that was higher than expected, supporting on-going surveillance efforts [2]. While the findings in this case report support the use of first-line TLD ART without baseline HIV resistance testing, further study is needed to understand the impact of transmitted or PrEP-acquired INSTI resistance on the response to INSTI-based ART in different populations and settings.

## Supplementary Material

ofaf645_Supplementary_Data

## Supplementary Data

Supplementary materials are available at *Open Forum Infectious Diseases* online. Consisting of data provided by the authors to benefit the reader, the posted materials are not copyedited and are the sole responsibility of the authors, so questions or comments should be addressed to the corresponding author.
