# Supplementary material for: Sustained HIV Suppression With Co-formulated Tenofovir Disoproxil Fumarate/Lamivudine/Dolutegravir in a Person With Transmitted Dolutegravir Resistance and Pretreatment Resistance to Lamivudine: a Case Report From HPTN 083
Source: Open Forum Infect Dis. 2025 Oct 15;12(11):ofaf645. doi: 10.1093/ofid/ofaf645 (PMC12575077; doi:10.1093/ofid/ofaf645)
Supplement: ofaf645_Supplementary_Data [file ofaf645_supplementary_data.pdf]

## SUPPLEMENTARY FILES

**Supplementary File 1. Laboratory methods** .......... 2
**Supplementary File 2. Results from the single-genome sequencing assay** .......... 4
**Supplementary File 3. HIV phenotyping results** .......... 5
**References** .......... 6

## Supplementary File 1. Laboratory methods

Laboratory testing was performed in real-time at the study site and retrospectively at the HIV Prevention Trials Network (HPTN) Laboratory Center (LC; Johns Hopkins Univ. School of Medicine) and the Univ. of Pittsburgh School of Medicine. Methods used for laboratory testing are described below.

### HIV Testing

Study visits in HPTN 083 were conducted at enrollment, four weeks later, and then every eight weeks. HIV screening was performed at each scheduled study visit and at interim visits if indicated. Methods used to determine HIV status and the timing of HIV acquisition in HPTN 083 are described previously.[1,2] HIV screening was performed using HIV rapid tests and a laboratory-based antigen/antibody (Ag/Ab) test. Additional testing was performed using locally available antibody and/or HIV RNA assays. Additional HIV testing was performed retrospectively at the HPTN LC using the following assays: the Architect HIV Ag/Ab Combo assay (Ag/Ab test; Abbott Diagnostics, Wiesbaden, Germany), the APTIMA HIV-1 RNA Qualitative assay (Hologic, Inc., San Diego, CA), the Geenius HIV 1/2 Supplemental Assay (Bio-Rad Laboratories, Inc., Hercules, CA), and the RealTime HIV-1 Viral Load Assay (Abbott Molecular, Des Plaines, IL, lower limit of quantification: 40 copies/mL).[3]

### HIV genotyping

HIV genotyping was performed retrospectively using stored plasma samples. Samples with viral loads >500 copies/mL were tested using the GenoSure PRIme assay (protease [PR], reverse transcriptase [RT], and integrase [IN] regions; Monogram Biosciences, South San Francisco, CA). This assay is based on next-generation sequencing and detects mixtures present at frequencies of 10% or higher.[4] Plasma samples with viral loads <500 copies/mL were tested at the University of Pittsburgh using a single-genome amplification and sequencing assay (IN region only; Sanger sequencing).[5,6] Identification of resistance-associated mutations (RAMs) and interpretation of HIV drug resistance was performed using the HIVdb algorithm[7] v9.6 from the Stanford HIV Drug Resistance Database.[8,9] Mutations recommended for the surveillance of transmitted drug resistance (SDRM) were identified using the World Health Organization (WHO) 2009 SDRM list and the WHO HIVResNet working group 2020 SDRM list.[10,11]

### HIV phenotyping

Recombinant donor-derived virus was generated by amplifying HIV PR, RT, and IN from plasma HIV RNA from a sample collected at the participant's first HIV-positive visit. The amplified DNA was bulk cloned into an HIV-1xxLAI vector, as previously described.[12] Donor-derived recombinant plasmids containing PR, RT, and IN sequences were used to transfect 293T cells using Lipofectamine 3000. Viral supernatant was collected 48 hours after transfection. Sanger sequencing was performed for the recombinant virus and the plasma virus. The genotypes were compared to confirm presence of RAMs in both viruses (nucleoside/nucleotide reverse transcriptase inhibitor RAMs: L74V, Y115F, M184V, K219N; non-nucleoside reverse transcriptase inhibitor RAMs K103R, G190E, V179I; and integrase strand transfer inhibitor RAMs L74I, T97A, G140S, and Q148H).

The laboratory-developed TZM-bl HIV-1 Phenotyping Assay was used to measure the susceptibility of the recombinant virus from the participant (donor-derived virus) to dolutegravir (DTG), lamivudine (3TC), tenofovir (TFV), bictegravir (BIC), and cabotegravir (CAB) as previously described.[12] TZM-bl cells are an indicator cell line that allows quantitative analysis of HIV-1 replication. TZM-bl cells were generated from HeLa cells that stably over-express CD4 and CCR5 and have separately integrated copies of the luciferase and ß-galactosidase genes under control

of the HIV-1 promoter. These cells naturally express CXCR4 receptors. TZM-bl cells were plated at 10,000 cells per well overnight. The cells were treated with non-toxic serial dilutions of each drug and were infected with a dilution of infectious HIV-1 wildtype (lab strain xxLAI)[13] or donor-derived virus normalized for the output of 140,000 relative light units (RLU) in virus control wells. After a 48-hour incubation at 37°C, cells were lysed, and luminescence was measured in RLU using a commercially available luciferase detection system (Britelite Plus; Revvity, Waltham, MA). Fifty and ninety percent inhibitory concentrations ($IC_{50}$; $IC_{90}$) were determined for DTG, 3TC, TFV, BIC, and CAB. The inhibitory concentration (IC) is a measure of a drug's antiviral potency and is traditionally based on *in vitro* assessments. The $IC_{50}$ refers to the half-maximal inhibitory concentration, or the concentration at which there is a 50% reduction in viral replication; the $IC_{90}$ is the concentration required to inhibit viral replication by 90%. Fold-change in drug susceptibility was calculated in comparison to wild-type HIVxxLAI virus. The starting concentration, dilution series, and number of concentrations tested for each drug are shown in the table below.

| Drug | Starting concentration | Dilution series | # concentrations tested |
|---|---|---|---|
| DTG | 1,852 nM | 3-fold | 13 |
| 3TC | 100 μM | 3-fold | 8 |
| TFV | 100 μM | 3-fold | 8 |
| BIC | 300 nM | 3-fold | 8 |
| CAB | 300 nM | 3-fold | 8 |

Abbreviations: 3TC: lamivudine; BIC: bictegravir; CAB: cabotegravir; DTG: dolutegravir; nM: nanomoles μM: micromoles; TFV: tenofovir.

HIV subtyping

HIV subtyping was performed by phylogenetic analysis at Monogram Biosciences.

Pharmacology testing

Concentrations of CAB, DTG and TFV in plasma and TFV-diphosphate (TFV-DP) in dried blood spots were measured using liquid chromatography-tandem mass spectrometry, as previously described.[3,14] The limits of quantification for the antiretroviral assays are as follows: CAB, 25 ng/mL; DTG, 100 ng/mL; TFV, 0.31 ng/mL; TFV-DP, 31.3 fmol per punch. TFV concentrations of 10 and 40 ng/mL correspond to 4 and 7 doses/week, respectively.[15] TFV-DP concentrations of 350, 700, and 1,250 fmol/punch correspond to 2, 4, and 7 doses/week, respectively.[16]

**Supplementary File 2. Results from the single-genome sequencing assay**

The table below shows the results obtained with the single-genome sequencing assay (integrase region) for samples collected when the participant was on antiretroviral treatment (see Figure 1A). This assay allows one to assess the linkage of resistance mutations. Major integrase strand transfer inhibitor (INSTI) resistance-associated mutations (RAMs) and INSTI accessory mutations were identified using the Stanford HIV Drug Resistance Database. Major RAMs are bolded. The numbers in parentheses indicate the number of sequences with the mutation(s).

| Study visit | Days since first pos visit | Viral load[a] | Total sequences generated | Major INSTI RAMs | INSTI accessory mutations |
|---|---|---|---|---|---|
| F/U Week 12 | 972 | 302 | 22 | **G140S**, **Q148H** (22) | L74I (22), T97A (22), A128T (1) |
| F/U Week 24 | 1,032 | 274 | 21 | **G140S**, **Q148H** (21) | L74I, T97A (21) |
| F/U Week 36 | 1,122 | 360 | 21 | **G140S**, **Q148H** (21) | L74I, T97A (21) |
| F/U Week 48 | 1,217 | <40 | 6 | **G140S**, **Q148H** (6) | L74I, T97A (6) |

Footnote:

[a] HIV viral load values were obtained at the HPTN Laboratory Center (RNA copies/mL). The lower limit of quantification (LLOQ) for this assay is 40 copies/mL. <40 indicates that HIV RNA was detected below the LLOQ.

Abbreviations:

F/U: Follow-up; INSTI: integrase strand transfer inhibitor; pos: (HIV) positive; RAMs: resistance-associated mutations.

## Supplementary File 3. HIV phenotyping results

The figure below shows dose-response curves from the TZM-bl HIV-1 Phenotyping Assay for donor-derived virus (recombinant virus containing protease, reverse transcriptase, and integrase DNA from the study participant, shown in blue) and the wild type reference virus (HIVxxLAI, shown in green). The X-axis shows the log of drug concentration and the Y-axis shows the percentage of inhibition of HIV replication.

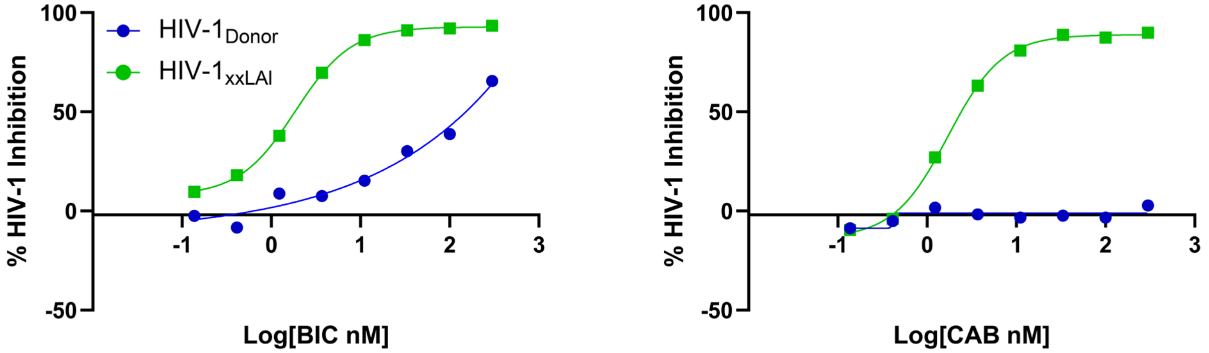


The tables below show the 50% and 90% inhibitory concentrations ($IC_{50}$ and $IC_{90}$ values) and fold-change values for resistance of the donor-derived virus and the wild type reference virus.

| **Drug** | **$IC_{50}$** (donor-derived HIV) | **$IC_{50}$** (reference, wild type HIV) | **$IC_{50}$ fold change** (donor/reference) |
|---|---|---|---|
| BIC | 143 nM[a] | 1.9 nM | 67 |
| CAB | >300 nM | 1.7 nM | >176 |

| **Drug** | **$IC_{90}$** (donor-derived HIV) | **$IC_{90}$** (reference, wild type HIV) | **$IC_{90}$ fold change** (donor/reference) |
|---|---|---|---|
| BIC | >300 nM[a] | 9.1 nM | >33 |
| CAB | >300 nM | 8.0 nM | >38 |

Footnote:

[a] The $IC_{50}$ and $IC_{90}$ values for BIC were calculated using simple linear regression of appropriate data points because accurate $IC_{50}$ and $IC_{90}$ determination by non-linear regression was not possible.

Abbreviations: BIC: bictegravir; CAB: cabotegravir; $IC_{50}$: *in vitro* 50% inhibitory concentration; IC90: *in vitro* 90% inhibitory concentration; nM: nanomolar.

<u>**References**</u>

1. Landovitz RJ, Donnell D, Clement ME, et al. Cabotegravir for HIV prevention in cisgender men and transgender women. N Engl J Med **2021**; 385:595-608.

2. Landovitz RJ, Hanscom BS, Clement ME, et al. Efficacy and safety of long-acting cabotegravir compared with daily oral tenofovir disoproxil fumarate plus emtricitabine to prevent HIV infection in cisgender men and transgender women who have sex with men 1 year after study unblinding: a secondary analysis of the phase 2b and 3 HPTN 083 randomised controlled trial. Lancet HIV **2023**; 10:e767-e778.

3. Marzinke MA, Grinsztejn B, Fogel JM, et al. Characterization of human immunodeficiency virus (HIV) infection in cisgender men and transgender women who have sex with men receiving injectable cabotegravir for HIV prevention: HPTN 083. J Infect Dis **2021**; 224:1581-1592.

4. Monogram Biosciences. GenoSure PRIme. https://monogrambio.labcorp.com/resources/genotyping/genosure-prime. Accessed 29 July 2025.

5. Halvas EK, Joseph KW, Brandt LD, et al. HIV-1 viremia not suppressible by antiretroviral therapy can originate from large T cell clones producing infectious virus. J Clin Invest **2020**; 130:5847-5857.

6. Eshleman SH, Fogel JM, Halvas EK, et al. HIV RNA screening reduces integrase strand transfer inhibitor resistance risk in persons receiving long-acting cabotegravir for HIV prevention. J Infect Dis **2022**; 226:2170-2180.

7. Liu TF, Shafer RW. Web resources for HIV type 1 genotypic-resistance test interpretation. Clin Infect Dis **2006**; 42:1608-1618.

8. Rhee SY, Gonzales MJ, Kantor R, Betts BJ, Ravela J, Shafer RW. Human immunodeficiency virus reverse transcriptase and protease sequence database. Nucleic Acids Res **2003**; 31:298-303.

9. Shafer RW. Rationale and uses of a public HIV drug-resistance database. J Infect Dis **2006**; 194:S51-58.

10. Bennett DE, Camacho RJ, Otelea D, et al. Drug resistance mutations for surveillance of transmitted HIV-1 drug-resistance: 2009 update. PLoS ONE **2009**; 4:e4724.

11. Tzou PL, Rhee SY, Descamps D, et al. Integrase strand transfer inhibitor (INSTI)-resistance mutations for the surveillance of transmitted HIV-1 drug resistance. J Antimicrob Chemother **2020**; 75:170-182.

12. Penrose KJ, Wallis CL, Brumme CJ, et al. Frequent cross-resistance to dapivirine in HIV-1 subtype C-infected individuals after first-line antiretroviral therapy failure in South Africa. Antimicrob Agents Chemother **2017**; 61:e01805-01816.

13. Shi C, Mellors JW. A recombinant retroviral system for rapid in vivo analysis of human immunodeficiency virus type 1 susceptibility to reverse transcriptase inhibitors. Antimicrob Agents Chemother **1997**; 41:2781-2785.

14. Rackow AR, Pandey A, Price AL, Marzinke MA. Rapid and sensitive liquid chromatographic-tandem mass spectrometric methods for the quantitation of dolutegravir in human plasma and breast milk. J Mass Spectrom Adv Clin Lab **2024**; 34:1-7.

15. Donnell D, Baeten JM, Bumpus NN, et al. HIV protective efficacy and correlates of tenofovir blood concentrations in a clinical trial of PrEP for HIV prevention. J Acquir Immune Defic Syndr **2014**; 66:340-348.

16. Grant RM, Anderson PL, McMahan V, et al. Uptake of pre-exposure prophylaxis, sexual practices, and HIV incidence in men and transgender women who have sex with men: a cohort study. Lancet Infect Dis **2014**; 14:820-829.
